# Assessment of online patient education material for eye cancers: A cross-sectional study

**DOI:** 10.1371/journal.pgph.0001967

**Published:** 2023-10-16

**Authors:** Courtney van Ballegooie, Jasmine Wen

**Affiliations:** 1 Experimental Therapeutics, BC Cancer, Vancouver, British Columbia, Canada; 2 Faculty of Medicine, University of British Columbia, Vancouver, British Columbia, Canada; 3 Department of Chemistry, Simon Fraser University, Burnaby, British Columbia, Canada; 4 Department of Science, University of Western Ontario, Western University, London, Ontario, Canada; The Australian National University, AUSTRALIA

## Abstract

The objective of this study was to assess online American patient education material (PEM) related to eye cancers in order to determine the quality of the content and appropriateness of the contents’ reading level as it relates to the American population. PEMs were extracted from fifteen American cancer and ophthalmology associations and evaluated for their reading level using ten validated readability scales. PEMs then had all words extracted and evaluated for their difficulty and familiarity. The quality of the PEMS were assessed according to DISCERN, Heath On the Net Foundation Code of Conduct (HONCode), and JAMA benchmarks. Overall, online PEMs from the associations were written at a 11^th^ grade reading level, which is above the recommended 6^th^ grade reading level. The difficult word analysis identified that 26% of words were unfamiliar. Only one of the fifteen association held a HONCode certification while no organization met the standards of all four JAMA benchmarks. The average score for DISCERN was 2.4 out of a total of 5 for the fifteen questions related to treatment option information quality. Consideration should be made to create PEMs at an appropriate grade reading level to encourage health literacy and ultimately promote health outcomes. Associations should also focus on incorporating easily identifiable quality indicators to allow patients to better identify reputable resources.

## 1. Introduction

The internet has become a widely used resource for health-related information due to its increasing popularity and accessibility. In fact, approximately 61% of Americans use the internet with the intent of finding information related to their health [1, 2]. This online health information seeking behavior has been accompanied by a significant increase in the number of available health-related information materials over the last few years. Therefore, it is imperative that quality health related information is easily identifiable, reliable, and written at a level that most Americans can understand to better inform and empower those in their health-related decision making [3].

Health literacy is defined as “the degree to which individuals have the ability to find, understand, and use information and services to inform health-related decisions and actions for themselves and others” [4]. In a study conducted by the National Assessment of Adult Literacy, it was found that 22% of American adults had basic health literacy levels and 14% had below basic health literacy levels [5]. Additionally, the average grade reading level (GRL) of Americans is between the 7^th^ and 8^th^ grade level [6]. Due to these findings, it is recommended that health related information should be written two GRLs below the average GRL (e.g., a 6^th^ GRL or below) to enable comprehension [4, 6, 7]. Clinically, patients who have low health literacy levels have difficulty understanding health information and are at an increased risk for negative health outcomes [8]. Readability is an important component of health literacy which refers to the ease with which an individual can understand written text and can be used to assess the GRL of health materials [9]. Online patient education materials (PEMs) that have low readability may create unnecessary barriers for patients seeking to understand health information. As patients increasingly turn towards the internet for health information, online PEMs need to be written at a 6^th^ GRL or below.

Cancer is the second leading cause of death in the United States, accounting for an estimated 1.9 million new cancer diagnoses and 609,360 deaths in 2022 [10]. For many cancer patients, the internet is one of the first resources they pursue to obtain cancer-related information. In fact, between 16–69% of patients have been found to search the internet to obtain cancer-related information [11]. Health literacy is particularly important in a cancer setting, where patients are exposed to numerous treatment options and are required to make decisions during multiple stages of cancer care such as prevention, screening, and treatment. As a result, poor health literacy may negatively affect patients at every stage of their cancer journey [12]. Unfortunately, there is a disparity in the amount of online health content that patients can obtain with regards to rare cancers, which are defined as those cancers that occur in less than 40,000 people per year, as compared to more common malignancies [13]. Many eye cancers are classified as rare, with an estimated 3,360 new cases of eye cancers in the United States in 2022 [14, 15]. Readability studies have been conducted on online patient education materials from a variety of topics in ophthalmology or cancer [16–20]. Some studies focus on a single ophthalmology condition, such as diabetic retinopathy, while other readability studies assess a wide range of ophthalmology conditions [6, 16]. Similarly, there have been readability studies focused on common cancers such as breast, colon, and prostate cancers [21]. To date, there have been no readability and quality assessments of online PEMs for rare eye cancers. Research pertaining to this is further necessitated by the fact that there are very limited online options for patients to obtain PEMs regarding these specific cancers. Therefore, this study aims to assess the readability and quality of online patient education materials on rare eye cancers.

## 2. Methods

### 2.1. Sample collection

In this study, we evaluated the websites of 20 major national ophthalmologic and cancer associations, including the American Academy of Ophthalmology (AAO), American Association of Ophthalmic Oncologists and Pathologists, American Association for Pediatric Ophthalmology and Strabismus (AAPOS), American Glaucoma Society (AGS), American Society of Cataract and Refractive Surgery, American Society of Ophthalmic, Plastic and Reconstructive Surgery, American Society of Retina Specialists, American Uveitis Society, Cornea Society, North American Neuro-Ophthalmology Society (NANOS), Aim at Melanoma, Melanoma Research Foundation (MRF), Ocular Melanoma Foundation (OMF), American Cancer Society (ACS), American Childhood Cancer (ACC), Cancer.net (C.net), CancerCare (CC), Children’s Oncology Group (COG), National Cancer Institute (NCI), and National Pediatric Cancer (NPC). Associations were selected to consider the range of individuals the information would be applicable to (e.g., national association were favored over state specific associations), geography (U.S. based), as well as through a literature search [7, 8, 17, 18]. These 20 associations were then evaluated to see if they contained information related to eye cancers. Fifteen of the 20 associations contained eye cancer related information (Table 1).

**Table 1:** Cancer and ophthalmology associations’ patient education material: A depiction of the associations that provide patient education material and their respected number of patient education material(s).

American Association	Documents, No.
Cancer.net (C.net)	56
American Cancer Society (ACS)	41
American Academy of Ophthalmology (AAO)	10
Ocular Melanoma Foundation (OMF)	8
Melanoma Research Foundation (MRF)	5
Children’s Oncology Group (COG)	4
National Cancer Institute (NCI)	3
American Society of Retina Specialists (ASRS)	2
CancerCare (CC)	2
American Childhood Cancer (ACC)	1
National Pediatric Cancer (NPC)	1
National Eye Institute	1
American Optometric Association	1
American Association of Pediatric Ophthalmology	1
Aim at Melanoma	1

A depiction of the American Cancer and Ophthalmology associations that provide patient education material and their respected number of unique patient education documents.

During May and June 2021, all internet-based PEMs were extracted from the associations’ websites. Fifteen national associations were identified that contained information related to eye cancers and are listed in Table 1 along with the number of unique PEMs obtained from each association. The PEMs included materials describing any eye cancer related topic with intended use by patients. If a document was a pdf, then they were manually converted to plain text for further analysis. Text sections of nonmedical information were removed from each of the PEMs before analysis, as described previously [22–26].

### 2.2. Document readability analysis

A readability assessment was then performed as described previously [22–26]. The software package Readability Studio professional edition version 2019.3 (Oleander Software, Ltd) was utilized to determine the GRL of the PEMs through eight numerical scales and two graphical scales. The eight numerical scales comprised of the Degrees of Reading Power (DRP) and Grade Equivalent (GE) test, Flesch-Kincaid Grade Level (FK), Simple Measure of Gobbledygook Index (SMOG), Coleman-Liau Index (CLI), Gunning Fog Index (GF), New Fog Count (NFC), New Dale-Chall (NDC), and Ford, Caylor, Sticht (FORCAST) scale. The two graphical scales included the Raygor Readability Estimate Graph (RREG) and the Fry Readability Graph (FRG). These ten scales are externally validated and are frequently used to evaluate the readability of medical text [22–26]. Multiple readability scales were utilized to provide a holistic understanding of the PEM’s readability and to ensure no single parameter skewed the GRL of any one association. Most readability scales use a combination of parameters, such as the average sentence or word length, the average number of words per sentence, the number of syllables per word, or the presence of difficult or unfamiliar words (S5 Table). Limitations can occur if only one readability scale is used. For example, PEMs which contain medical jargon with few syllables, such as biopsy, may have a low GRL using a read- ability scale which relies on the number of syllables (e.g., FORCAST), but would have a high GRL in NDC which looks at the relative amount of unfamiliar to familiar words.

**Fig 1:**
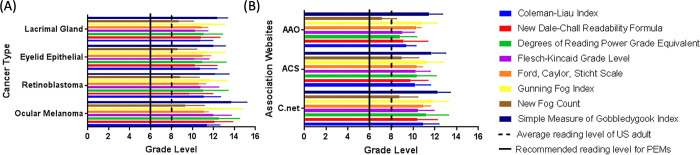
Depicts a compilation of the numerical readability analyses: Degrees of Reading Power and Grade Equivalent test, Flesch-Kincaid Grade Level, Simple Measure of Gobbledygook Index, Coleman-Liau Index, Gunning Fog Index, New Fog Count, New Dale-Chall, Ford, Caylor, Sticht (FORCAST) for **(a)** each of the eye cancer types which contained more than 10 patient education materials (PEMS) and **(b)** the top three PEM contributing associations including Cancer.net (C.net), the American Cancer Society (ACS), and the American Academy of Ophthalmology (AAO).

PEMs often contain text that must be modified to ensure that the readability scales can be applied properly during the analysis. The alterations overcome limitations in the analysis where long stings of bullet points containing no punctuation will be seen as a single run on sentence despite the known increase in comprehension bullet points have. To address this limitation, PEMs were individually edited to create high- and low-sentence documents, as described previously [22–26]. The GRL using the eight numerical scales organized by cancer type and the top three contributing associations can be seen in Fig 1A and 1B respectively. Greater granularity of each of the readability scales is provided in S1 Fig. The FRG and RREG assessment of each cancer type and association can be seen in S2 and S3 Figs respectively, and reports the results of the high sentence estimates (equating to the lowest potential GRL).

### 2.3. Difficult word analysis

The difficult word analysis was implemented as described previously [22–25]. Analysis included the identification of the number and percent of complex words (three or more syllable words), long (six or more characters) words, and unfamiliar words according to the NDC criteria [S1–S4 Tables]. All words from the PEMs were also extracted and compared to the NDC word list as well as the New General Service List. Words that appeared in either of the lists, including words with the same base word but different tense, were removed and considered as non-jargon words. All words that appeared in less than three PEMs or had a total frequency below three were excluded from analysis. The top ten most frequently identified words were then extracted and had their different tenses combined. Alternative words were then proposed for these most frequently identified words, either using the Readability Studio Software, the Merriam-Webster Thesaurus or in consultation with a medical doctor, to identify synonyms that can decrease the difficulty of the word.

### 2.4. Quality analysis

A quality analysis was performed using three well established, validated tools including Health On the Net (HON) Foundation Code of Conduct (HONCode), DISCERN, and JAMA benchmarks. HON is a non-profit organization which holds a consultative status with the Economic and Social Council of the United Nations. HONCode evaluates the credibility and reliability of information for medical and health websites. Websites can apply for certification and are assessed for disclosure of authors’ qualifications, attribution/citation of sources, data protection, justifiability, transparency, and disclosure of sources of funding and advertising. At the time that this manuscript was written, over 7,000 websites hold a HONCode certification with 80% of the websites located in the United States [27]. All associations had their HONCode certification status identified through the HONCode portal. DISCERN, as seen in S6 Table, assesses the quality and reliability of consumer health information by grading 16 items (concerning reliability, description of treatment choices, and overall rating) from 1 (inferior) to 5 (superior). Quality is assessed on a scale from 16 to 80, with higher scores indicating higher quality information [28]. Associations had their treatment related PEMs collected to exhaustion and analyzed by two independent reviewers according the DISCERN criteria. The scores of the two reviewers were then averaged for each of the PEMs and the association’s score was determined by averaging all of the PEMs analyzed. Lastly, the JAMA benchmarks were used to assess the accountability of each website. This instrument evaluates the presence of four components: authorship, references, disclosure (including ownership, advertising policy, sponsorship, and conflicts of interests), and currency (e.g. date of creation/update) [29]. Up to ten PEMs were randomly collected from each association and evaluated by two independent reviewers for each of the four criteria. Because the scores for the JAMA benchmarks are binary, 1 (meets all criteria) and 0 (does not meet all criteria), the mode was used to determine the score of each of the associations.

### 2.5. Statistical methods

Graphical data in Fig 1 was reported as the arithmetic mean with the error bars representing the standard deviation. Briefly, data sets had their normality tested using a Shapiro-Wilk test and were cross-examined using a quantile-quantile plot when central limit theorem conditions were not met. Equal variance was tested using a Bartlett’s test to see if the data would need to be transformed before analysis. Normally distributed data with equal variance then underwent a one-way Analysis of Variance (ANOVA). If the data was not normally distributed, then a non-parametric, Kruskal-Wallis test, was employed. Multiple comparison’s tests were utilized to identify differences between sample means in the ANOVA analysis. Statistics were analyzed using Graph Pad Prism 9.

## 3. Results

### 3.1. Readability analysis

137 PEMs were collected and analyzed (Table 1). The average GRL of the eight readability scales for each cancer type are as follows: ocular melenoma (11.9 +/- 1.6), retinoblastoma (10.3 +/- 1.5), eyelid epithelial cancer (10.6 +/- 1.7), and lacrimal glad cancer (10.8 +/- 1.3). The overall mean was (10.9 +/- 1.5), with a GRL range from 5 to 17. When individual PEMs had their eight readability scores averaged, none were below a 6^th^ GRL and 2 (2%) of the 106 PEMs were below an 8^th^ grade level. The RREG (S3A–S3D Fig) ranges from a 6^th^ GRL to a grade level equivalent to that of a university professor with 10% and 0% of the PEMs exhibiting a grade level below eight and six GRL respectively. The FRG (S3A–S3D Fig) ranges from a 6^th^ grade to a 17^th^ (university educated) reading level with 7% of the PEMs exhibiting a grade level below eight and 0% below six. Fig 1A illustrates a summary of the readability tests for each of the different cancer types as well as the top three contributing opthamology and oncology associations in Fig 1B.

The average GRL of the eight readability scales for the top three contributing associations are as follows: Cancer.net (C.net) (10.8 +/- 1.4), American Cancer Society (ACS) (10.3 +/- 1.4), and American Academy of Ophthalmology (AAO) (9.5 +/- 1.4). The overall mean was (10.2 +/- 1.4), with a GRL range from 4 to 17. When individual PEMs had their eight readability scores averaged, none were below a 6^th^ GRL and 3 (3%) of the 107 PEMs were below an 8^th^ GRL. The RREG (S3E–S3G Fig) ranges from a 6^th^ GRL to a grade level equivalent to that in university with 12% and 0% of the PEMs exhibiting a GRL below eight and six level respectively. The FRG (S2E–S2G Fig) ranges from a 6^th^ grade to a 17^th^ (university educated) reading level with 10% of the PEMs exhibiting a grade level below eight and 0% below six. No cancer type nor association was identified as being statistically significantly more difficult or easy as any of the other cancer types or associations (defined as having a statistical significance on five or more of the eight numerical scales relative to its comparitors).

### 3.2. Difficult word analysis

From the difficult word analysis, it was found that the PEMs, on average, were comprised of (17.4 +/- 5.1) %, (14.3 +/- 3.4) %, (13.7 +/- 3.1) %, (15.3 +/- 3.5) % complex words for PEMs pertaining to ocular melanoma, retinoblastoma, eyelid epithelial cancer, and lacrimal gland cancer respectively (S2 Table). Additionally, (35.1 +/- 5.0) %, (33.0 +/- 4.4) %, (35.0 +/- 4.5) %, and (33.6 +/- 4.1) % of words were identified to contain 6 or more characters and (30.0 +/- 5.2) %, (24.1 +/- 5.2) %, (25.2 +/- 4.8) %, and (27.2 +/- 5.2) % of words were identified as unfamiliar, respectively. The top three associations which contributed the most PEMs (C.net, ACS, and AAO) had similar values for complexity [(14.8 +/- 3.2) %, (12.3 +/- 3.2) %, and (14.6 +/- 2.4) % respectively], length [(34.6 +/- 4.2)%, (31.0 +/- 3.3) %, and (31.5 +/- 2.6) % respectively], and unfamiliarity [(25.7 +/- 4.8)%, (23.3 +/- 4.3) %, and (24.3 +/- 5.0) % respectively]. There were no associations nor cancer type which was identified as significantly less difficult than any other (S3 and S4 Tables). The most frequent terms included melanoma (-s), retinoblastoma (-s), radiation, diagnose (-ed, -is), chemotherapy, retina (-l), and metastases (-is, -ed, -tic) (S1 Table). 94% of the top terms identified were medical jargon.

### 3.3. Quality analysis

**Fig 2:**
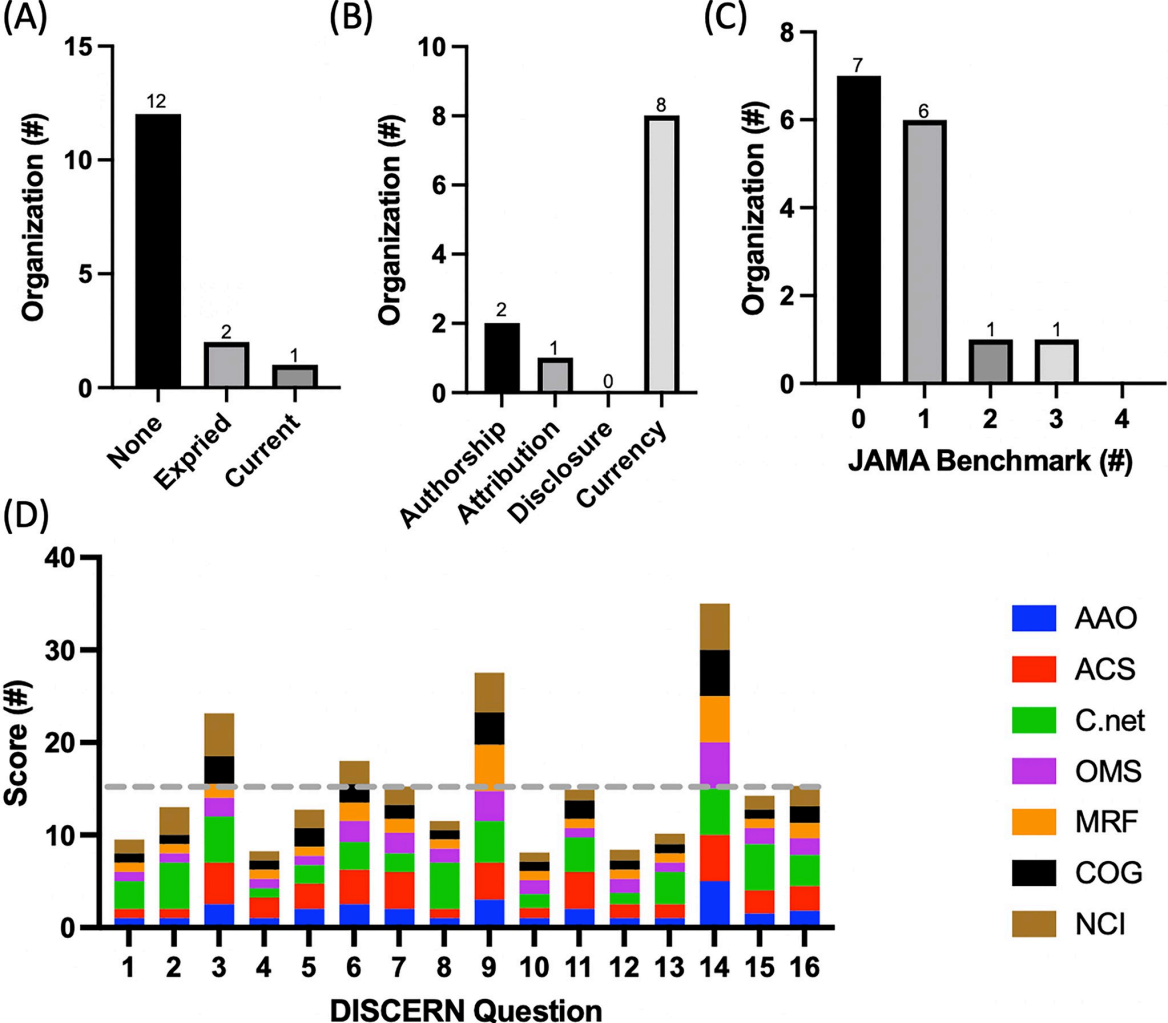
Depicts a compilation of the quality analyses: Heath On the Net Foundation Code of Conduct (HONCode), JAMA benchmarks, and DISCERN. **(a)** the number of associations which have either never had a HONCode certification, has an expired HONCode certification, or holds a current HONCode certification **(b)** the number of organizations that display the quality indicators of JAMA benchmarks **(c)** the number of organizations which display zero, one, two, three, or all four of the JAMA benchmarks **(d)** the average score for each of the associations for each DISCERN question. Grey dashed line indicates average score.

Only C.net held an up-to-date HONCode certification with the National Eye Institute and the ACS being the only other associations that had ever held a HONCode certification (Fig 2A). It was identified that currency was the most common JAMA benchmark (displayed by eight associations) while disclosure was the least common, with zero of the association displaying all of the required disclosure criteria (Fig 2B). Additionally, no association displayed all four JAMA benchmarks while the majority of the associations displayed either one or zero of the benchmarks (Fig 2B). Only seven of the fifteen organizations contained PEMs which discussed treatment options and, could therefore be evaluated using the DISCERN criteria. The average score was 2.4 out of 5 with C.net and the ACS receiving the highest average score per question, 3.4 and 2.7 respectively, and the Melanoma Research Foundation receiving the lowest average score per question of 1.7. The first eight questions of DISCERN are meant to assess if the source of information is reliable while the next seven questions assess the quality of the information regarding treatment choices. On average, associations scored more poorly on reliability (2.0) than (2.4) quality (Fig 2D). Specifically, associations scored above average on questions pertaining to relevance, presence of unbiases, treatment mechanism, and the display of multiple treatment options (3.3, 2.8, 3.9, and 5 respectively). Questions pertained to having clearly defined aims, displayed sources of information, presented treatment benefits, and the discussion of what would happen if no treatment was utilized, however, scored the lowest (1.4, 1.2, 1.2, and 1.2 respectively).

## 4. Discussion

Associations providing online cancer PEMs must take readability into consideration, especially for patients with low literacy levels. Readability assessments of online PEMs on various medical conditions and topics have been conducted [30–34]. Those related to cancer and ophthalmology have also been examined; however, this is the first study evaluating the readability and quality of online PEMs for rare eye cancers [6, 16, 17, 35–44]. Previous readability studies in the medical field, including topics in ophthalmology and cancer, show that online PEMs are written above the average GRL of Americans. This study is in line with previous findings, showing that the GRL of rare eye cancer PEMs are written above the recommended 6^th^ GRL.

Many readability formulas have been designed to evaluate health education texts, such as the SMOG, FRG, and NDC formulas, however, the most commonly used formulas are FK and FRES [45, 46]. Formulas such as the SMOG and FRG have been endorsed nationally by the National Cancer Institute and the Centers for Disease Control and Prevention [46]. Due to differences in readability formula calculations, there is variation in readability estimates when different formulas are applied to the same text [45]. There is no gold standard readability formula in healthcare; however, using more than one formula may improve the validity of the results when the average GRL is used [45, 46].

On average, the GRL for different cancer types was found to be approximately an 11^th^ GRL and the average GRL for the top three associations was approximately a 10^th^ GRL. In addition, the difficult word analysis identified that approximately 26% of words were unfamiliar and that 94% of the top terms were medical jargon. A study that evaluated PEMs from similar ophthalmology associations used in this study such as AAO, American Association for Pediatric Ophthalmology and Strabismus, and the American Society of Retina Specialists also found PEMs to be written above recommended reading levels, with the average for each association being above a 10^th^ GRL [17]. These findings, in combination with the difficult word and readability analysis performed here, suggests that many associations should be focusing on decreasing the difficulty of their text by replacing unfamiliar words, most of which are medical jargon, and long words. Tailored recommendations of which words should be considered for modification to better enable comprehension for each cancer type and top three PEM contributing associations can be found in S1 Table.

The quality assessments used in this study are also commonly used together to evaluate healthcare related PEMs [41–44, 47]. DISCERN and JAMA benchmarks are both validated instruments, while HONCode has been active since 1995, with more than 7,300 websites certified across 102 countries [43]. HONCode certification, which evaluates the reliability and credibility of websites, was only present for C.net. In other studies, it was found that between a 6 to 41% of the health care websites examined were HONCode certified [41–44, 47]. Some reasons for the few HONCode certifications identified in this study could include 1) a possible lack of awareness by designers of health information websites, 2) the presence of an incurred cost for 3^rd^ party certification, and 3) an awareness or concern over the lengthy certification process, which can take up to 14 weeks [42, 43]. When assessing for the JAMA benchmarks, which measure accountability, none of the associations met all four benchmarks. This is in alignment with other studies that showed low adherence to the accountability criteria [16, 41]. Other studies only found one or two websites meeting all JAMA benchmarks [42, 47, 48]. Interestingly, while there was variability in the percent of websites that adhered to each of the benchmarks across studies, the majority of studies identified currency as the most commonly adhered to benchmark. While there is no way to know with certainty without engaging the individual organizations, the currency attribution may be the most common due to 1) the ease of gathering / reporting and 2) their lower potential liability relative to other benchmarks. While this may be the case, reporting of all the benchmarks is essential as authorship affiliations, commercial disclosures, and other factors can influence patient’s decisions [41, 42, 44, 47, 48].

Scores for DISCERN varied depending on question, with the average score per question being 2.4 out of 5 for the seven associations that discussed treatment options. Associations performed above average on questions involving relevance, presence of unbiases, multiple treatment options, and treatment mechanism, while questions involving defined aims, treatment benefits, no treatment, and sources of information received a score below average. These findings provide insight into specific website content that could be improved, which would result in more well-rounded eye cancer PEMs. Previous readability studies that use DISCERN show varied scores, with certain areas scoring higher than others. DISCERN assessments on breast and colorectal cancer found scores to be “good” and thyroid cancer scores to be “fair,” while oral dysplasia received an average score of 2.24 out of 5, similar to rare eye cancers [42–44]. Interestingly, other studies also found DISCERN criteria such as presence of unbiases, relevance, and description of treatment mechanisms to receive higher scores, while questions involving consequences of no treatment and quality of life received lower scores [41, 43, 44]. Concepts such as consequences of no treatment and impact of treatment on quality of life take multiple considerations into account. Given the sensitivity and complexity of the topics, these conversations are perhaps better to be discussed with a health care team [44]. Supplemental to this, a section within treatment related PEMs could include a description of how to hold conversations with the healthcare team surrounding palliation or quality of life concerns.

Although multiple readability formulas were used to determine GRL, there are limitations. The readability formulas in this study only measure data such as sentence length and structure, number of syllables per word, or number of familiar words. They do not measure other factors such as document organization, layout, style, content, visuals, font size, color, and motivation of the reader, which influence document comprehension [45, 46]. Additionally, this study did not take cultural sensitivity into account, which could impact readability [49]. Next, the literacy levels of eye cancer patients may be different from that of the general American population. For example, the average age of diagnosis for choroidal melanomas is 60 years old, representing an older patient population [50]. It has been found that adults aged 65 and older are the largest group with health literacy skills of “below basic” [51]. It should also be taken into consideration that this study only involved the general United States population and PEMs were only retrieved from 15 American-based cancer and ophthalmology associations; therefore, this study is not generalizable to other populations and results from the assessments may not apply to other rare eye cancer PEMs on the internet.

Many resources have provided suggestions to improve the readability of online PEMs. The Centers for Disease Control and Prevention created a guide for designing health communication materials while the National Institutes of Health designed *Clear & Simple*, a guide to help health communicators create appropriate health content for people with low health literacy levels [52, 53]. Other readability studies recommend shortening sentence and word length, using an active voice, having a clear webpage layout, including multimedia, and changing medical jargon to simpler words [32, 35]. Additional strategies to improve readability could involve physicians discussing PEMs with patients and PEM creators obtaining feedback from rare eye cancer patients. It is acknowledged that implementing these recommendations will demand significant effort and time from both designers of health information websites and healthcare providers. Additionally, it will likely necessitate supplementary financial investments from the respective associations. Despite these perceived costs, the benefits of ensuring that patients possess sufficient knowledge, skills, and confidence to effectively manage chronic diseases can result in higher levels of patient activation. This, in turn, has been associated with increased screening rates and improved survival in various types of cancer [54–57].

## 5. Conclusion

Overall, this study demonstrates that online PEMs for rare eye cancers are written above the GRL of the general American population and above the recommended 6^th^ GRL. Many websites were found to lack quality measures according to HONCode, JAMA benchmarks, and DISCERN criteria. Rare eye cancer associations should revise online PEMs to be written at an appropriate GRL and display appropriate quality measures to ensure that PEMs are easily identifiable and accessible to patients.

## Supporting information

S1 Fig Box plot displaying the grade level determined by each readability measure for each cancer type and the top three contributing associations. (DOCX)

S2 Fig Fry Readability Graph assessment of all high sentence estimate online patient education materials for each cancer type and the top three contributing associations. (DOCX)

S3 Fig Raygor Readability Estimate Graph of all high sentence estimate online patient education materials for each cancer type and the top three contributing associations. (DOCX)

S1 Table Difficult words with alternative word recommendations for each cancer type and the top three contributing associations. (DOCX)

S2 Table Difficult words analysis for each cancer type and the top three contributing associations. (DOCX)

S3 Table Difficult words analysis statistics for each cancer type. (DOCX)

S4 Table Difficult words analysis statistics for of the top three contributing associations. (DOCX)

S5 Table Readability formulas. (DOCX)

S6 Table DISCERN instrument criteria. (DOCX)

S7 Table Examples of PEMs that were evaluated to have an average high (>6th) or low (≤6th) GRL. (DOCX)

S1 Data Data for readability, difficult word, and quality analyses. (XLSX)

## Abbreviations

PEM: Patient Education Material
DRP GE: Degrees of Reading Power and Grade Equivalent test
FK: Flesch-Kincaid Grade Level
SMOG: Simple Measure of Gobbledygook Index
CLI: Coleman-Liau Index
GF: Gunning Fog Index
NFC: New Fog Count
NDC: New Dale-Chall
FORCAST: Ford, Caylor, Sticht scale
RREG: Raygor Readability Estimate Graph
FRG: Fry Readability Graph
FRES: Flesch Reading Ease Score
GRL: grade reading level
HONCode: Health On the Net Foundation Code of Conduct
AAO: American Academy of Ophthalmology
ACS: American Cancer Society
C.net: Cancer.net

## References

[1] Pew Research Center. 2021 April 7 [cited 25 Aug 2022]. Internet/Broadband Fact Sheet [Internet]. Washington: Pew Research Centre. Available from: https://www.pewresearch.org/internet/fact-sheet/internet-broadband/

[2] Pew Research Centre. 2009 June 11 [cited 25 Aug 2022]. The Social Life of Health Information [Internet]. Washington: Pew Research Centre. Available from https://www.pewresearch.org/internet/2009/06/11/the-social-life-of-health-information/

[3] McMullan M. Patients using the Internet to obtain health information: how this affects the patient–health professional relationship. Patient Educ and Couns. 2006 Oct;63(1−2): 24−8. doi: 10.1016/j.pec.2005.10.006 16406474

[4] Centers for Disease Control and Prevention. Reviewed 2021 Feb 2 [cited 25 Aug 2022]. What Is Health Literacy? [Internet]. Georgia: Centers for Disease Control and Prevention. Available from: https://www.cdc.gov/healthliteracy/learn/index.html

[5] Kutner M, Greenburg E, Jin Y, Paulsen C. The Health Literacy of America’s Adults: Results from the 2003 National Assessment of Adult Literacy. National Center for education statistics. 2006. Available from: https://nces.ed.gov/pubs2006/2006483.pdf

[6] Edmunds MR, Barry RJ, Denniston AK. Readability assessment of online ophthalmic patient information. JAMA Ophthalmol. 2013 Dec 1;131(12): 1610–6. doi: 10.1001/jamaophthalmol.2013.5521 24178035

[7] Hutchinson N, Baird GL, Garg M. Examining the Reading Level of Internet Medical Information for Common Internal Medicine Diagnoses. Am J Med. 2016 Jun;129(6): 637–9. doi: 10.1016/j.amjmed.2016.01.008 26829438

[8] Walsh TM, Volsko TA. Readability assessment of internet-based consumer health information. Respir Care. 2008 Oct;53(10): 1310–5. 18811992

[9] Mcinnes N, Haglund BJ. Readability of online health information: implications for health literacy. Inform Health Soc Care. 2011 Dec;36(4): 173–89. doi: 10.3109/17538157.2010.542529 21332302

[10] American Cancer Society. 2022 [cited 25 Aug 2022]. Cancer Facts & Figs 2022 [Internet]. Georgia: American Cancer Society. Available from: https://www.cancer.org/content/dam/cancer-org/research/cancer-facts-and-statistics/annual-cancer-facts-and-figures/2022/2022-cancer-facts-and-figures.pdf

[11] Maddock C, Lewis I, Ahmad K, Sullivan R. Online information needs of cancer patients and their organizations. Ecancermedicalscience. 2011;5. doi: 10.3332/ecancer.2011.235 22276067 PMC3239170

[12] Koay K, Schofield P, Jefford M. Importance of health literacy in oncology. Asia Pac J Clin Oncol. 2012 Mar;8(1): 14–23. doi: 10.1111/j.1743-7563.2012.01522.x 22369440

[13] National Cancer Institute. 2019 Feb 27 [cited 25 Aug 2022]. About Rare Cancers [Internet]. Bethesda: National Cancer Institute;. Available from: https://www.cancer.gov/pediatric-adult-rare-tumor/rare-tumors/about-rare-cancers

[14] American Cancer Society. Revises 2022 Jan 12 [cited 25 Aug 2022]. Key Statistics for Eye Cancer [Internet]. Georgia: American Cancer Society. Available from: https://www.cancer.org/cancer/eye-cancer/about/key-statistics.html

[15] MD Anderson Cancer Centre. [cited 25 Aug 2022]. Eye cancer [Internet]. Houston: MD Anderson Cancer Center. Available from: https://www.mdanderson.org/cancer-types/eye-cancer.html

[16] Kloosterboer A, Yannuzzi NA, Patel NA, Kuriyan AE, Sridhar J. Assessment of the quality, content, and readability of freely available online information for patients regarding diabetic retinopathy. JAMA Ophthalmol. 2019 Nov 1;137(11): 1240–5. doi: 10.1001/jamaophthalmol.2019.3116 31436789 PMC6707011

[17] Huang G, Fang CH, Agarwal N, Bhagat N, Eloy JA, Langer PD. Assessment of online patient education materials from major ophthalmologic associations. JAMA Ophthalmol. 2015 Apr 1;133(4): 449–54. doi: 10.1001/jamaophthalmol.2014.6104 25654639

[18] Agarwal N, Hansberry DR, Sabourin V, Tomei KL, Prestigiacomo CJ. A comparative analysis of the quality of patient education materials from medical specialties. JAMA Intern Med. 2013 Jul;173(13): 1257–9. doi: 10.1001/jamainternmed.2013.6060 23689468

[19] Fefer M, Lamb CC, Shen AH, Clardy P, Muralidhar V, Devlin PM, et al. Multilingual analysis of the quality and readability of online health information on the adverse effects of breast cancer treatments. JAMA Surg. 2020 Aug;155(8): 781–4. doi: 10.1001/jamasurg.2020.1668 32520317 PMC7287949

[20] Perni S, Rooney MK, Horowitz DP, Golden DW, McCall AR, Einstein AJ, et al. Assessment of use, specificity, and readability of written clinical informed consent forms for patients with cancer undergoing radiotherapy. JAMA Oncol. 2019 Aug;5(8): e190260 doi: 10.1001/jamaoncol.2019.0260 31046122 PMC6499131

[21] Friedman DB, Hoffman-Goetz L, Arocha JF. Readability of cancer information on the internet. J Cancer Educ. 2004 Jun;19(2): 117–22. doi: 10.1207/s15430154jce1902_13 15456669

[22] Man A, van Ballegooie C. Assessment of the Readability of Web-Based Patient Education Material From Major Canadian Pediatric Associations: Cross-sectional Study. JMIR Pediatr Parent. 2022 Mar 16;5(1): e31820. doi: 10.2196/31820 35293875 PMC8968558

[23] Hoang PM, van Ballegooie C. Assessment of the Readability and Quality of Online Patient Education Material for Chronic Medical Conditions. Healthcare. 2022; 10(2):234. doi: 10.3390/healthcare10020234 35206850 PMC8872454

[24] van Ballegooie C, Hoang P. Health Services: A Mixed Methods Assessment of Canadian Cancer Patient Education Materials Related to the 2019 Novel Coronavirus. Cancer Control. 2021 Feb 5;28. 10.1177/1073274821989709 PMC8482715 33563050

[25] van Ballegooie C, Hoang P. Assessment of the readability of online patient education material from major geriatric associations. J Am Geriatr Soc. 2021 Apr;69(4): 1051–6. doi: 10.1111/jgs.16960 33236778

[26] van Ballegooie C. Assessment of Canadian patient education material for oncology pharmaceutics. J Oncol Pharm Pract. 2021 Oct;27(7):1578–87. doi: 10.1177/1078155220960823 33019874

[27] Wrigley Kelly NE, Murray KE, McCarthy C, O’Shea DB. An objective analysis of quality and readability of online information on COVID-19. Health Technol. 2021 Sep;11(5): 1093–9. doi: 10.1007/s12553-021-00574-2 34189011 PMC8222704

[28] Charnock D, Shepperd S, Needham G, Gann R. DISCERN: an instrument for judging the quality of written consumer health information on treatment choices. J Epidemiol Community Health. 1999 Feb 1;53(2): 105–11. doi: 10.1136/jech.53.2.105 10396471 PMC1756830

[29] Silberg WM, Lundberg GD, Musacchio RA. Assessing, controlling, and assuring the quality of medical information on the Internet: Caveant lector et viewor—Let the reader and viewer beware. JAMA. 1997 Apr 16;277(15): 1244–5. 9103351

[30] Kher A, Johnson S, Griffith R. Readability assessment of online patient education material on congestive heart failure. Adv Prev Med. 2017 Jun. doi: 10.1155/2017/9780317 28656111 PMC5471568

[31] Rhee RL, Von Feldt JM, Schumacher HR, Merkel PA. Readability and suitability assessment of patient education materials in rheumatic diseases. Arthritis Care Res. 2013 Oct;65(10): 1702–6. doi: 10.1002/acr.22046 23687011

[32] Eloy JA, Li S, Kasabwala K, Agarwal N, Hansberry DR, Baredes S, et al. Readability assessment of patient education materials on major otolaryngology association websites. OTO Open. 2012 Nov;147(5): 848–54. doi: 10.1177/0194599812456152 22864405

[33] D’Alessandro DM, Kingsley P, Johnson-West J. The readability of pediatric patient education materials on the World Wide Web. Arch Pediatr Adolesc Med. 2001 Jul 1;155(7): 807–12. doi: 10.1001/archpedi.155.7.807 11434848

[34] De Oliveira GS Jr, Jung M, Mccaffery KJ, McCarthy RJ, Wolf MS. Readability evaluation of Internet-based patient education materials related to the anesthesiology field. J Clin Anesth. 2015 Aug 1;27(5): 401–5. doi: 10.1016/j.jclinane.2015.02.005 25912728

[35] AlKhalili R, Shukla PA, Patel RH, Sanghvi S, Hubbi B. Readability assessment of internet-based patient education materials related to mammography for breast cancer screening. Acad Radiol. 2015 Mar 1;22(3): 290–5. doi: 10.1016/j.acra.2014.10.009 25488695

[36] Pruthi A, Nielsen ME, Raynor MC, Woods ME, Wallen EM, Smith AB. Readability of American online patient education materials in urologic oncology: a need for simple communication. Urology. 2015 Feb 1;85(2): 351–6. doi: 10.1016/j.urology.2014.10.035 25623686 PMC4308671

[37] Tian C, Champlin S, Mackert M, Lazard A, Agrawal D. Readability, suitability, and health content assessment of web-based patient education materials on colorectal cancer screening. Gastrointest Endosc. 2014 Aug 1;80(2): 284–90. doi: 10.1016/j.gie.2014.01.034 24674352

[38] Weiss KD, Vargas CR, Ho OA, Chuang DJ, Weiss J, Lee BT. Readability analysis of online resources related to lung cancer. J Surg Res. 2016 Nov 1;206(1): 90–7. doi: 10.1016/j.jss.2016.07.018 27916381

[39] Samuel D, Vilardo N, Isani SS, Kuo DY, Gressel GM. Readability assessment of online gynecologic oncology patient education materials from major governmental, non-profit and pharmaceutical organizations. Gynecol Oncol. 2019 Sep 1;154(3):616–21. doi: 10.1016/j.ygyno.2019.06.026 31324452

[40] Dobbs T, Neal G, Hutchings HA, Whitaker IS, Milton J. The readability of online patient resources for skin cancer treatment. Oncol Ther. 2017 Dec;5(2): 149–60.

[41] Doubleday AR, Novin S, Long KL, Schneider DF, Sippel RS, Pitt SC. Online information for treatment for low-risk thyroid cancer: assessment of timeliness, content, quality, and readability. J Cancer Educ. 2021 Aug;36(4): 850–7. doi: 10.1007/s13187-020-01713-5 32108292 PMC11014722

[42] Alsoghier A, Riordain RN, Fedele S, Porter S. Web-based information on oral dysplasia and precancer of the mouth–quality and readability. Oral Oncol. 2018 Jul 1;82: 69–74. doi: 10.1016/j.oraloncology.2018.05.003 29909904

[43] Grewal P, Alagaratnam S. The quality and readability of colorectal cancer information on the internet. Int J Surg. 2013 Jun 1;11(5): 410–3. doi: 10.1016/j.ijsu.2013.03.006 23523948

[44] Nghiem AZ, Mahmoud Y, Som R. Evaluating the quality of internet information for breast cancer. Breast. 2016 Feb 1;25: 34–7. doi: 10.1016/j.breast.2015.10.001 26547835

[45] Wang LW, Miller MJ, Schmitt MR, Wen FK. Assessing readability formula differences with written health information materials: application, results, and recommendations. Res Social Adm Pharm. 2013 Sep 1;9(5): 503–16. doi: 10.1016/j.sapharm.2012.05.009 22835706

[46] Badarudeen S, Sabharwal S. Assessing readability of patient education materials: current role in orthopaedics. Clin Orthop Relat Res. 2010 Oct;468(10): 2572–80. doi: 10.1007/s11999-010-1380-y 20496023 PMC3049622

[47] Mozafarpour S, Norris B, Borin J, Eisner BH. Assessment of readability, quality and popularity of online information on ureteral stents. World J Urol. 2018 Jun;36(6): 985–92. doi: 10.1007/s00345-018-2179-9 29435639

[48] Tran J, Tsui E. Assessment of the readability, availability, and quality of online patient education materials regarding uveitis medications. Ocul Immunol and Inflamm. 2020 Apr 12: 1–6. doi: 10.1080/09273948.2020.1737144 32275173

[49] Wilson FL, Racine E, Tekieli V, Williams B. Literacy, readability and cultural barriers: critical factors to consider when educating older African Americans about anticoagulation therapy. J Clin Nurs. 2003 Mar;12(2): 275–82. doi: 10.1046/j.1365-2702.2003.00711.x 12603561

[50] Maheshwari A, Finger PT. Cancers of the eye. Cancer Metastasis Rev. 2018 Dec;37(4): 677–90. doi: 10.1007/s10555-018-9762-9 30203109

[51] Centers for Disease Control and Prevention. 2009 [cited 25 Aug 2022]. Improving Health Literacy for Older Adults: Expert Panel Report 2009 [Internet]. Georgia: Centers for Disease Control and Prevention. Available from: https://www.cdc.gov/healthliteracy/pdf/olderadults-508.pdf

[52] Centers for Disease Control and Prevention. 2009 April [cited 25 Aug 2022]. Simply put; a guide for creating easy-to-understand materials [Internet]. Georgia: Centre for Disease Control and Prevention. Available from: https://stacks.cdc.gov/view/cdc/11938

[53] National Institutes of Health. Reviewed 2021 July 7 [cited 25 Aug 2022]. Clear & Simple [Internet]. Bethesda: National Institutes of Health. Available from: https://www.nih.gov/institutes-nih/nih-office-director/office-communications-public-liaison/clear-communication/clear-simple

[54] Hibbard JH, Stockard J, Mahoney ER, Tusler M. Development of the Patient Activation Measure (PAM): Conceptualizing and Measuring Activation in Patients and Consumers. Health Serv Res. 2004;39: 1005–1026. doi: 10.1111/j.1475-6773.2004.00269.x 15230939 PMC1361049

[55] Katz ML, Fisher JL, Fleming K, Paskett ED. Patient Activation Increases Colorectal Cancer Screening Rates: A Randomized Trial among Low-Income Minority Patients. Cancer Epidemiol Biomark Prev. 2012;21: 45–52. 10.1158/1055-9965.EPI-11-0815 PMC3650905 22068288

[56] Papadakos JK, Giannopoulos E, McBain S, Forbes L, Jain P, Samoil D, et al. Quality Assessment of Cancer Patient Education Materials: The Current State of Systemic Therapy Patient Education in Fourteen Cancer Centres across Ontario, Canada. Support. Care Cancer 2021;29: 3513–3519. doi: 10.1007/s00520-020-05859-2 33151399

[57] van Ballegooie C, Heroux D, Hoang P, Garg S. Assessing the Functional Accessibility, Actionability, and Quality of Patient Education Materials from Canadian Cancer Agencies. Curr Oncol. 2023;30: 1439–1449. doi: 10.3390/curroncol30020110 36826071 PMC9955234

