# Supplementary material for: Assessment of online patient education material for eye cancers: A cross-sectional study
Source: PLOS Glob Public Health. 2023 Oct 16;3(10):e0001967. doi: 10.1371/journal.pgph.0001967 (PMC10578596; doi:10.1371/journal.pgph.0001967)
Supplement: S1 Fig — (DOCX) [file pgph.0001967.s001.docx]

**
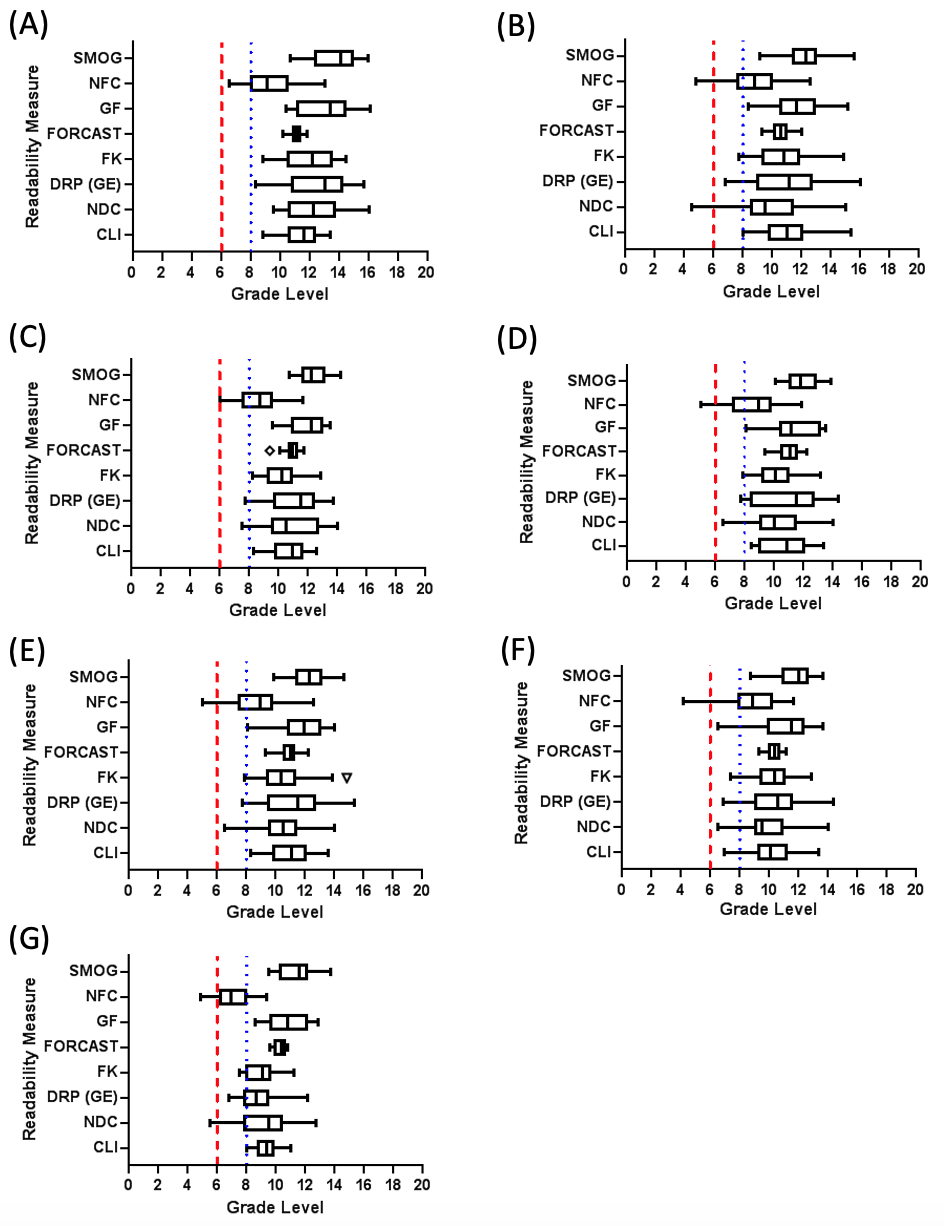
**

**S1 Fig:** Box plots displaying the grade level determined by each readability measure for each cancer type and the top three contributing associations. The readability measures include the Degrees of Reading Power (DRP) and Grade Equivalent (GE) test, Flesch-Kincaid Grade Level (FK), Simple Measure of Gobbledygook Index (SMOG), Coleman-Liau Index (CLI), Gunning Fog Index (GF), New Fog Count (NFC), New Dale-Chall (NDC), and Ford, Caylor, Sticht (FORCAST) scale. Grade levels determined by the eight readability scales pertaining to: (a) ocular melanoma, (b) retinoblastoma, (c) lacrimal gland cancer, (d) eyelid epithelial cancer, (e) Cancer.net, (f) The American Cancer Society, and (g) The American Academy of Ophthalmology.
