# Supplementary material for: Assessment of online patient education material for eye cancers: A cross-sectional study
Source: PLOS Glob Public Health. 2023 Oct 16;3(10):e0001967. doi: 10.1371/journal.pgph.0001967 (PMC10578596; doi:10.1371/journal.pgph.0001967)
Supplement: S2 Fig — (DOCX) [file pgph.0001967.s002.docx]

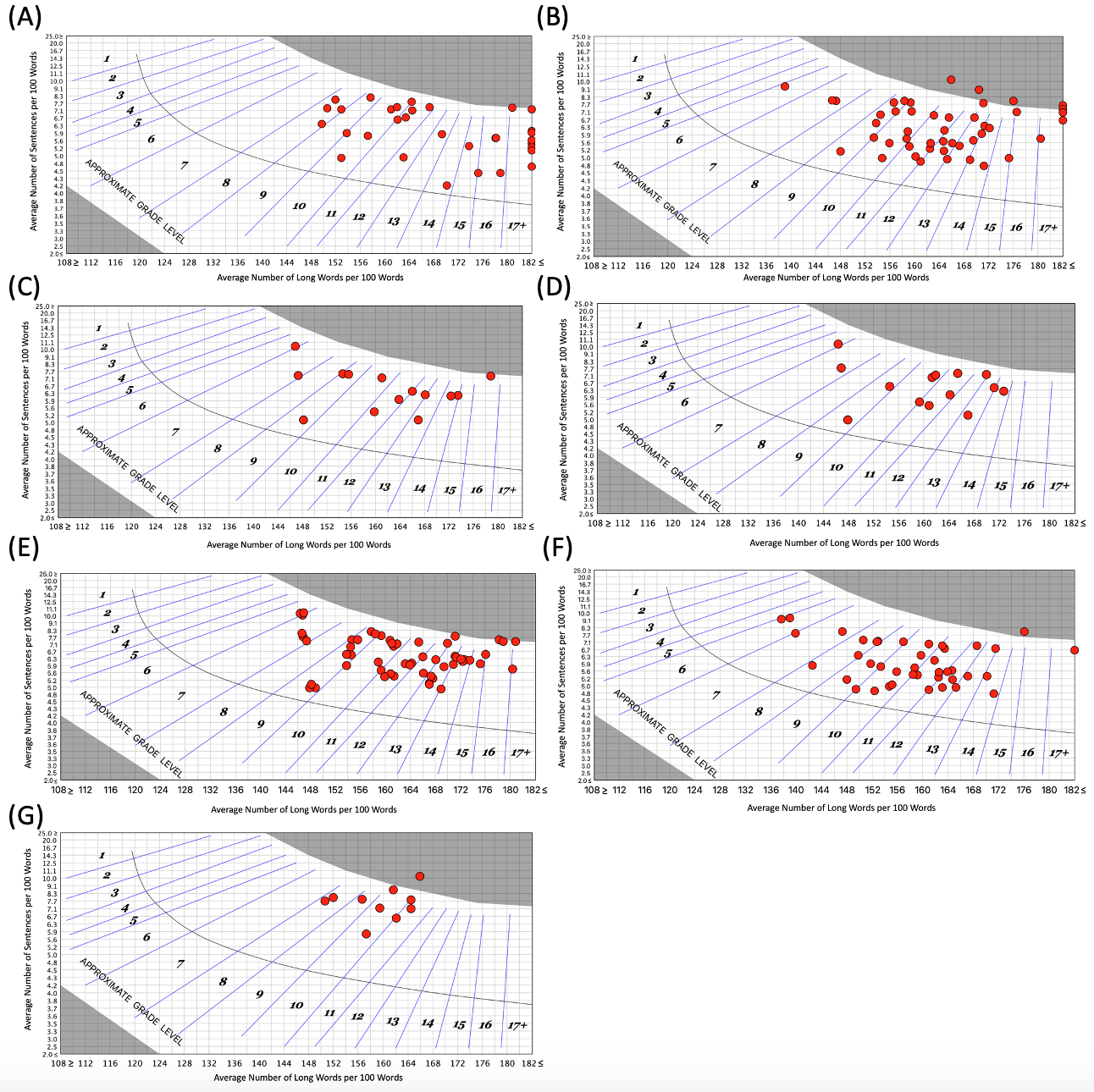


**S2 Fig:** Fry Readability Graph (FRG) assessment of all high sentence estimate online patient education materials for each cancer type and the top three contributing associations. (a) Ocular melanoma FRG, (b) Retinoblastoma FRG, (c) Lacrimal gland cancer FRG, (d) Eyelid epithelial cancer FRG, (e) Cancer.net FRG, (f) The American Cancer Society FRG, and (g) The American Academy of Ophthalmology FRG.
