# Supplementary material for: Assessment of online patient education material for eye cancers: A cross-sectional study
Source: PLOS Glob Public Health. 2023 Oct 16;3(10):e0001967. doi: 10.1371/journal.pgph.0001967 (PMC10578596; doi:10.1371/journal.pgph.0001967)
Supplement: S3 Fig — (DOCX) [file pgph.0001967.s003.docx]

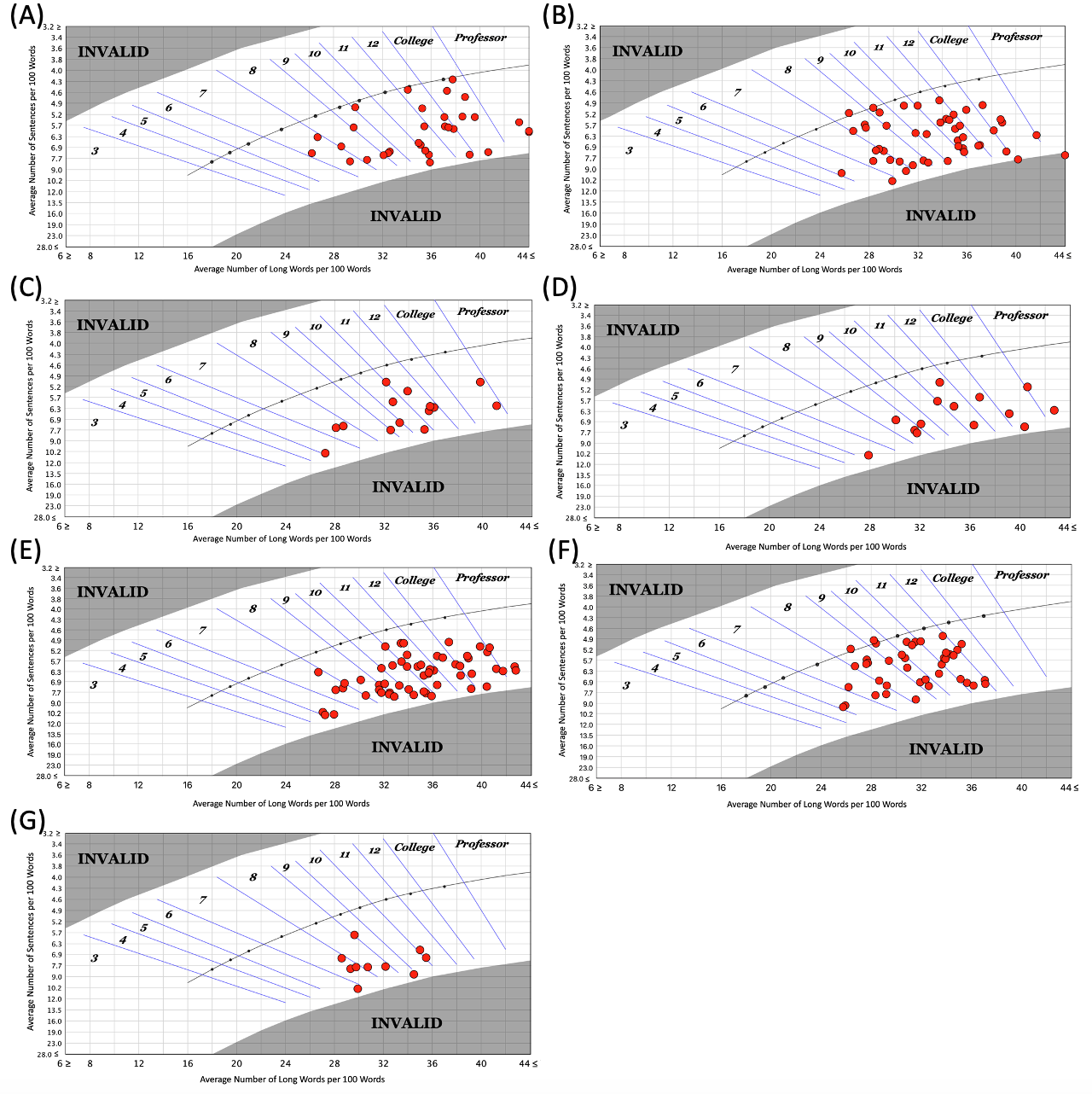


**S3 Fig:** Raygor Readability Estimate Graph (RREG) of all high sentence estimate online patient education materials for each cancer type and the top three contributing associations. (a) Ocular melanoma RREG, (b) Retinoblastoma RREG, (c) Lacrimal gland cancer RREG, (d) Eyelid epithelial cancer RREG, (e) Cancer.net RREG, (f) The American Cancer Society RREG, and (g) The American Academy of Ophthalmology RREG.
