# Supplementary material for: Assessment of online patient education material for eye cancers: A cross-sectional study
Source: PLOS Glob Public Health. 2023 Oct 16;3(10):e0001967. doi: 10.1371/journal.pgph.0001967 (PMC10578596; doi:10.1371/journal.pgph.0001967)
Supplement: S1 Table — (DOCX) [file pgph.0001967.s004.docx]

| **S1 Table. Difficult Words with Alternative Word Recommendations** | | | | |
| --- | --- | --- | --- | --- |
| **Cancer Type or Association** | | **Difficult Word*** | **Frequency** | **Alternatives***** |
| Ocular melanoma | | | | |
|  | Melanoma (-s) | | 441 | Cancer; skin cancer |
|  | Ocular | | 127 | Eye |
|  | Radiation | | 125 | X-Ray; UV; treatment |
|  | Ciliary | | 108 | Cilia; supports eye lens |
|  | Intraocular | | 102 | In the eye |
|  | Metastases (-is, -ed, -tic) | | 85 | Cancer spread |
|  | Diagnose (-ed, -is) | | 76 | Detect |
|  | Conjunctiva (-l) | | 51 | Mucus layer; lining of the eyelid |
|  | Choroidal | | 43 | Colored blood vessel area; blood vessels of the eye |
|  | Retina (-l) | | 42 | A part in the back of the eye; part of the eye that captures light |
| Retinoblastoma | | | | |
|  | Retinoblastoma (-s) | | 601 | Cancer; eye cancer; retinal cancer |
|  | Radiation | | 147 | X-Ray; UV; treatment |
|  | Chemotherapy | | 145 | Chemo; drug; treatment |
|  | Retina (-l) | | 94 | A part in the back of the eye |
|  | Diagnose (-ed, -is) | | 90 | Detect |
|  | Heritable | | 50 | From parent to child |
|  | Mutation (-s) | | 36 | Change; gene change |
|  | Enucleation | | 31 | Removal of the eye |
|  | Cryotherapy | | 29 | Cold treatment; Cold therapy |
|  | Intraocular | | 28 | In the eye |
| Lacrimal gland cancer | | | | |
|  | Lacrimal | | 68 | Bone; helps produce tears; tear area |
|  | Radiation | | 34 | X-Ray; UV; treatment |
|  | Diagnose (-ed, -is) | | 33 | Detect |
|  | Lymphoma | | 18 | Cancer; blood cancer |
|  | Chemotherapy | | 15 | Chemo; drug; treatment |
|  | Palliative | | 14 | Focus on pain relief; end of life plan |
|  | Epithelial | | 14 | Skin; cell |
|  | Recurrence | | 13 | Come back; appear again; return |
|  | Survivorship | | 11 | Life after; living beyond |
|  | Malignant | | 10 | Cancer causing; causing harm or death |
| Eyelid epithelial cancer | | | | |
|  | Radiation | | 36 | X-Ray; UV; treatment |
|  | Diagnose (-ed, -is) | | 28 | Detect |
|  | Carcinoma | | 26 | Cancer; skin cancer |
|  | Biopsy | | 15 | Remove; take a sample |
|  | Palliative | | 14 | Focus on pain relief; end of life plan |
|  | Melanoma | | 13 | Cancer; skin cancer |
|  | Survivorship | | 11 | Life after; living beyond |
|  | Sebaceous | | 8 | Oil; fat; gland |
|  | Encourage (-ed) | | 8 | Support; cheer on |
|  | Cosmetic | | 6 | Look (-s); beauty |
| Cancer.net | | | | |
|  | Radiation | | 149 | X-Ray; UV; treatment |
|  | Diagnose (-ed, -is) | | 146 | Detect |
|  | Retinoblastoma (-s) | | 82 | Cancer; eye cancer; retinal cancer |
|  | Lacrimal | | 77 | Bone; helps produce tears; tear area |
|  | Chemotherapy | | 69 | Chemo; drug; treatment |
|  | Metastases (-is, -ed, -tic) | | 68 | Cancer spread |
|  | Palliative | | 56 | Focus on pain relief; end of life plan |
|  | Survivorship | | 54 | Life after; living beyond |
|  | Recurrence | | 50 | Come back; appear again |
|  | Intraocular | | 46 | In the eye |
| The American Cancer Society | | | | |
|  | Retinoblastoma (-s) | | 262 | Cancer; eye cancer; retinal cancer |
|  | Melanoma (-s) | | 184 | Cancer; skin cancer |
|  | Radiation | | 144 | X-Ray; UV; treatment |
|  | Diagnose (-ed, -is) | | 54 | Detect |
|  | Chemotherapy | | 48 | Chemo; drug; treatment |
|  | Retina (-l) | | 39 | A part in the back of the eye |
|  | Heritable | | 35 | From parent to child |
|  | Ciliary | | 31 | Cilia; supports eye lens |
|  | Uvea (-l) | | 30 | Part of the eye that provides nutrients; a layer in the eye |
|  | Biopsy | | 27 | Remove |
| The American Academy of Ophthalmology | | | | |
|  | Melanoma (-s) | | 44 | Cancer; skin cancer |
|  | Ocular | | 16 | Eye |
|  | Radiation | | 16 | X-Ray; UV; treatment |
|  | Retinoblastoma | | 14 | Cancer; eye cancer; retinal cancer |
|  | Ophthalmologist | | 13 | Eye doctor |
|  | Retina (-l) | | 7 | A part of the eye that receives light |
|  | Uvea | | 6 | Part of the eye that provides nutrients; a layer in the eye |
|  | Chemotherapy | | 6 | Chemo; drug; treatment |
|  | Artificial | | 5 | Handmade; machine made; fake |
| *Inclusion criteria for a “difficult word”: 1) Any word with ≥3 syllables that was used at least once in ≥3 patient education material and; 2) was either unlisted on the New Dale Chal list of familiar words and the New General Service List; **Alternatives selected are those that are considered synonymous while decreasing the individual word(s) syllable and/or character count. | | | | |

**S1 Table:** Difficult Words with Alternative Word Recommendations: Top 10 most frequent words by cancer type and the top three contributing associations that were considered complex, either by syllabic count, character count, of unfamiliarity as described by the difficult word analysis.
