# Supplementary material for: Assessment of online patient education material for eye cancers: A cross-sectional study
Source: PLOS Glob Public Health. 2023 Oct 16;3(10):e0001967. doi: 10.1371/journal.pgph.0001967 (PMC10578596; doi:10.1371/journal.pgph.0001967)
Supplement: S2 Table — (DOCX) [file pgph.0001967.s005.docx]

| **S2 Table. Difficult Words Analysis** | | | |
| --- | --- | --- | --- |
| **Cancer Type or Association** | **% 3+ Syllable Words** | **% 6+ Character words** | **% Unfamiliar Word*** |
| Ocular Melanoma | 17.37 (+/– 5.09) | 35.14 (+/– 4.98) | 29.67 (+/– 5.21) |
| Retinoblastoma | 14.29 (+/– 3.35) | 33.00 (+/– 4.45) | 24.09 (+/– 5.18) |
| Lacrimal gland cancer | 15.29 (+/– 3.48) | 33.56 (+/– 4.06) | 27.21 (+/– 5.17) |
| Eyelid epithelial cancer | 13.74 (+/– 3.12) | 34.93 (+/– 4.54) | 25.19 (+/– 4.82) |
| Cancer.net | 14.81 (+/– 3.25) | 34.58 (+/– 4.25) | 25.73 (+/– 4.76) |
| The American Cancer Society | 12.30 (+/– 3.18) | 31.02 (+/– 3.34) | 23.34 (+/– 4.29) |
| The American Academy of Ophthalmology | 14.47 (+/– 2.39) | 31.51 (+/– 2.58) | 24.34 (+/– 4.96) |
| *% Unfamiliar words as determined by the New Dale-Chall criteria. All analyses are reported as the mean and standard deviation (in brackets). | | | |

**S2 Table:** Difficult words analysis displaying the mean and standard deviation of the % 3+ syllable words, % 6+ character words, and % unfamiliar words found in the patient education material (PEMs) of each of the cancer types and top three contributing associations.
