# Supplementary material for: Assessment of online patient education material for eye cancers: A cross-sectional study
Source: PLOS Glob Public Health. 2023 Oct 16;3(10):e0001967. doi: 10.1371/journal.pgph.0001967 (PMC10578596; doi:10.1371/journal.pgph.0001967)
Supplement: S3 Table — (DOCX) [file pgph.0001967.s006.docx]

| **S3 Table. Difficult Word Analysis Statistics by Cancer Type** | | | | | | | | | | | | | | | |  |
| --- | --- | --- | --- | --- | --- | --- | --- | --- | --- | --- | --- | --- | --- | --- | --- | --- |
|  | **P Value** |  |  |  |  |  |  | |  |  | |  |  |  |  |  |
|  |  | **Pairwise Comparison of Ocular Melanoma to Other Eye Cancer Types** | | | | | | | | | | | | | |  |
| **Difficulty Analysis** | **Across All PEMs** | **Retinoblastoma** | | | | | | **Lacrimal gland cancer** | | | **Eyelid epithelial cancer** | | | |  |  |
| 3+ Syllables | 0.0162 | 0.0051 | | | | | | ns | | | 0.0255 | | | |  |  |
| 6+ Characters | ns | ns | | | | | | ns | | | ns | | | |  |  |
| Unfamiliar | <0.0001 | <0.0001 | | | | | | ns | | | 0.0380 | | | |  |  |
| *﻿P values for comparison across the different cancer types’ PEMs determined using analysis of variance (ANOVA). P values for pairwise comparisons between ﻿PEMs was determined using the Tukey’s test for difficulty scores with significant difference (P < .05) across the forms on ANOVA. Note: ns indicated non-significance. | | | | | | | | | | | | | | | | |

**S3 Table:** Analysis of variance (ANOVA) and pairwise comparison of the difficult words analyses [e.g. the % 3+ syllable words, % 6+ character words, and % unfamiliar words found in the patient education material (PEMs) of each of the cancer types].
