# Supplementary material for: Assessment of online patient education material for eye cancers: A cross-sectional study
Source: PLOS Glob Public Health. 2023 Oct 16;3(10):e0001967. doi: 10.1371/journal.pgph.0001967 (PMC10578596; doi:10.1371/journal.pgph.0001967)
Supplement: S5 Table — (DOCX) [file pgph.0001967.s008.docx]

| **S5 Table. Readability Formulas** |
| --- |
| **Coleman-Liau Index (CLI):** 0.0588 x (average number of letters per 100 words) – 0.296 x (average number of sentences per 100 words) –15.8  **Degrees of Reading Power (DRP)***: 100 – 100*(0.886593 - 0.083640 x (Number of characters / Number of words) + 0.161911 x ( Number of familiar Dale-Chall words / Number of words)³ – 0.021401 x (Number of words / Number of sentences) + .000577 x ( Number of words / Number of sentences)² – .000005*( Number of words / Number of sentences)³)  **Flesch-Kincaid Grade Level (FK):** 0.39 x (total number of words / total number of sentences) + 11.8 (total number of syllables / total number of words) – 15.59  **Ford, Caylor, Sticht (FORCAST):** 20-([Number of monosyllabic words per 150 words]/10)  **Fry Readability Graph (FRG):** a graph plotting the average number of syllables per 100 words on the x-axis and the average number of sentences per 100 words on the y-axis  **Gunning Fog Index (GF):** 0.4 x ([number of words]/[number of sentences]+(([number of complex words]/[number of words])*100))  **New Dale-Chall (NDC):** 0.1479 x (number of difficult words / number of words x 100) + 0.0496 x (number of words / number of sentences)  **New Fog Count (NFC):** (number of easy words + (3 x number of complex words) / (number of senesces) – 3) / 2  **Simple Measure of Gobbledygook Index (SMOG):** 1.0430 x √(number of polysyllables x [30 / number of sentences]) + 3.1291  **Raygor Readability Estimate Graph (RREG):** a graph plotting the average number of 6+ characters per 100 words on the x-axis and the average number of sentences per 100 words on the y-axis |
| *The grade equivalent (GE) of DRP has a corresponding GE according to their DRP score |

**S5 Table:** Readability formulas of the 8 numerical and 2 graphical readability tests

|  |
| --- |
