# Supplementary material for: Assessment of online patient education material for eye cancers: A cross-sectional study
Source: PLOS Glob Public Health. 2023 Oct 16;3(10):e0001967. doi: 10.1371/journal.pgph.0001967 (PMC10578596; doi:10.1371/journal.pgph.0001967)
Supplement: S6 Table — (DOCX) [file pgph.0001967.s009.docx]

| **S6 Table.** DISCERN Instrument Criteria |
| --- |

| 1 | Are the aims clear? |
| --- | --- |
| 2 | Does it achieve its aims? |
| 3 | Is it relevant? |
| 4 | Is it clear what sources of information were used to compile the publication? |
| 5 | Is it clear when the information used or reported was produced? |
| 6 | Is it balanced and unbiased? |
| 7 | Does it provide details of additional sources of support and information? |
| 8 | Does it refer to areas of uncertainty? |
| 9 | Does it describe how each treatment works? |
| 10 | Does it describe the benefits of each treatment? |
| 11 | Does it describe the risks of each treatment? |
| 12 | Does it describe what would happen if no treatment was used? |
| 13 | Does it describe how the treatment choices affect overall quality of life? |
| 14 | Is it clear that there is more than one treatment choice? |
| 15 | Does it provide support for shared decision making? |
| 16 | Overall rating |

**S6 Table.** DISCERN instrument criteria assessment of the quality and reliability of consumer health information pertaining
to treatment choice.
