# Supplementary material for: Assessment of online patient education material for eye cancers: A cross-sectional study
Source: PLOS Glob Public Health. 2023 Oct 16;3(10):e0001967. doi: 10.1371/journal.pgph.0001967 (PMC10578596; doi:10.1371/journal.pgph.0001967)
Supplement: S7 Table — (DOCX) [file pgph.0001967.s010.docx]

| **S7 Table. Examples of PEMs that were evaluated to have an average High (>6^th^) or Low (≤6^th^) GRL** | |
| --- | --- |
| Example patent education material (PEM) that had an average (across readability scales) low readability score (e.g., approximately ≤6^th^ grade reading level)*:  Questions to Ask About Retinoblastoma  It’s important to have honest, open discussions with your child’s doctors. You should ask any question, no matter how small it might seem. Here are some examples.  If retinoblastoma has just been diagnosed  How sure are you that my child has retinoblastoma?  Is only one eye affected or are there tumors in both eyes?  Do we know if this is the heritable (hereditary) form of retinoblastoma? How can we find out? If it is, what would this mean?  Has the cancer spread outside the eye?  What is the stage of the cancer, and what does that mean?  Has my child’s vision been affected?  Do we need any other tests before we can decide on treatment?  How much experience do you have treating this type of cancer?  Do we need to see any other types of doctors?  Who else will be on the treatment team, and what do they do?  When deciding on a treatment plan  What are our treatment options?  Can my child’s sight be saved? If so, how much?  What do you advise for treatment and why?  Should we get a second opinion? How do we do that? Can you recommend a doctor or cancer center?  Are there any clinical trials we should consider?  How soon do we need to start treatment?  How long will treatment last? What will it be like? Where will it be done?  What should we do to be ready for treatment?  What are the risks and side effects of the suggested treatments?  Which side effects start shortly after treatment and which ones might develop later on?  Will treatment affect the growth of the area around my child’s eye?  Will treatment affect my child’s ability to grow and develop?  Could treatment affect my child’s ability to have children later on?  During and after treatment  Once treatment begins, you’ll need to know what to expect and what to look for. Not all of these questions may apply, but getting answers to the ones that do may be helpful.  How will we know if the treatment is working?  Is there anything we can do to help manage side effects?  What symptoms or side effects should we tell you about right away?  How can we reach you or someone on your team on nights, weekends, or holidays?  Who can we talk to if we have questions about costs, insurance coverage, or social support?  What are the chances of the cancer coming back after treatment? What are our options if this happens?  Is there any risk of this type of tumor occurring in our other children or relatives?  Should we consider genetic counseling and testing?  What type of follow-up will my child need after treatment?  Does my child have a higher long-term risk of other cancers?  Along with these sample questions, be sure to write down some of your own. For instance, you might want more information about recovery times so you can plan your schedules. You may also want to ask about nearby or online support groups, where you may be able to get in touch with other families who have been through similar situations.  Also keep in mind that doctors aren't the only ones who can give you information. Other health care professionals, such as nurses and social workers, may be able to answer some of your questions. You can find out more about speaking with your health care team in The Doctor-Patient Relationship.  The grade reading level of the sample text above using the 8 numerical readability scales are as follows: DRP GE (5.1 GRL), FK (4.9 GRL), SMOG (8.1 GRL), CLI (7.2 GRL), GF (6.6 FRL), NFC (4.4 GRL), NDC (5.5 GRL), FORCAST (9.3 FRL) | |
| Example patent education material (PEM) that had an average (across readability scales) high readability score (e.g., >6^th^ grade reading level)*:  What’s New in Retinoblastoma Research?  Over the past few decades, research into retinoblastoma has led to many advances in treatment, which in turn has led to much higher cure rates and fewer side effects. Still, not all children are cured, and even those who are cured might still have long-term side effects from treatment, so more research is needed.  Research on retinoblastoma is being done at many medical centers, university hospitals, and other institutions around the world.  Genetics, genetic testing, and gene therapy  The defective gene responsible for nearly all retinoblastomas (the RB1 gene) was identified in 1986. This discovery, along with technical advances in finding DNA changes, has made genetic testing for heritable (hereditary) retinoblastoma possible.  A great deal of research has gone into figuring out how certain DNA changes in retinal cells cause them to become cancerous. Scientists understand these changes better for retinoblastoma than for most other cancer types. Although probably still years away, researchers hope that this understanding will one day lead to gene therapies, very specific treatments that can repair or counteract these DNA changes.  For example, researchers have found that an oncogene known as SYK is overactive in retinoblastoma cells. Drugs that target the protein this gene makes are now being developed. Another gene called MDM4 also seems to be involved in the development of retinoblastoma, and drugs aimed at blocking its effects are being studied.  Researchers have also found that a very small portion of retinoblastomas don’t seem to have changes in the RB1 gene, but instead have too many copies of another gene called MYCN. These cancers seem to be different in some important ways from those with RB1 gene changes.  Treatment  Research is building on the progress made in treating retinoblastoma over the past few decades.  Radiation therapy  External radiation therapy can be used to treat retinoblastoma, but it can cause side effects because the radiation often reaches nearby tissues as well. This is especially important in children with hereditary retinoblastoma, whose cells are more likely to be damaged by radiation.  Newer forms of radiation therapy such as intensity modulated radiation therapy (IMRT) and proton beam therapy can better target the tumor and spare nearby normal tissues. These techniques, which are described in Radiation Therapy for Retinoblastoma, may help doctors limit the side effects from radiation therapy.  Focal treatments  Doctors continue to improve the techniques used for cryotherapy, laser therapy (photocoagulation and thermotherapy), and other focal treatments. The goal is to kill tumor cells more precisely while sparing other parts of the eye.  Chemotherapy  Chemotherapy (chemo) has played a larger role in treating many retinoblastomas in recent years.  Systemic chemo: Chemo given into a vein (IV) is now commonly used to shrink tumors before local treatments such as cryotherapy or laser therapy. Chemo is also given to some children after the removal of the eye (known as adjuvant chemotherapy) to help prevent the recurrence of retinoblastoma outside the eye. Doctors are also studying the use of different chemo drugs, as well as new ways of combining currently used drugs, to try to improve how well chemo works.  Localized chemo: Doctors continue to improve upon newer ways of getting chemo into the eye, such as intra-arterial chemo and intravitreal chemo. These approaches let doctors get higher doses of chemo to the tumors while reducing many of the typical chemo side effects, and are quickly becoming part of the standard treatment for many retinoblastomas. These techniques are described in Chemotherapy for Retinoblastoma.  High-dose chemotherapy and stem cell transplant: A stem cell transplant (SCT) lets doctors give higher doses of chemo than could safely be given otherwise. (In the past, this type of treatment was commonly referred to as a bone marrow transplant.) Doctors are studying the use of this type of treatment in children with retinoblastoma that has spread outside the eye and who are unlikely to be cured with other treatments.  Chemo drugs can affect quickly dividing cells like those in the bone marrow, which is where new blood cells are made. Even though higher doses of these drugs might be more effective in treating tumors, they can’t be given because they would cause severe damage to bone marrow cells, leading to life-threatening shortages of blood cells.  To try to get around this problem, the doctor may treat the child with high-dose chemo (sometimes along with radiation therapy) and then use a stem cell transplant to “rescue” the bone marrow.  To learn more about stem cell transplants, including how they are done, see Stem Cell Transplant for Cancer.  Oncolytic virus therapy  Researchers are also trying to find ways to take advantage of the gene changes in retinoblastoma cells to treat these tumors. One example is VCN-01, a virus that’s been modified in the lab to infect and destroy cells that don’t have working copies of the RB1 gene (which includes the vast majority of retinoblastomas). This treatment is now being studied in the earliest phases of clinical trials.  The grade reading level of the sample text above using the 8 numerical readability scales are as follows: DRP GE (14.4 GRL), FK (12.9 GRL), SMOG (13.5 GRL), CLI (13.1 GRL), GF (13.5 FRL), NFC (11.1 GRL), NDC (11.5 GRL), FORCAST (11.2 FRL) | |
| *The “Low” grade reading level example was extracted from the American Cancer Society titled ”Questions to Ask About Retinoblastoma” and was the high sentence variation (e.g., the lowest possible GRL for the document)  ** The “High” grade reading level example was extracted from the American Cancer Society titled “What’s New in Retinoblastoma Research?” and was the average of the two sentence variations | |

| **S7 Table:** Examples of PEMs that were evaluated to have an average High (>6th) or Low (≤6th) grade reading level |
| --- |
